# Enhancing Intergenerational Programs: Older Adults’ Perspectives on Challenges and Opportunities

**DOI:** 10.1093/geroni/igaf122.3478

**Published:** 2025-12-31

**Authors:** Zeyu Liu, Rana Zadeh, Karl Pillemer

**Affiliations:** Cornell University, Jersey City, New Jersey, United States; Cornell University, Ithaca, New York, United States; Cornell University, Ithaca, New York, United States

## Abstract

Intergenerational programs (IGPs) are critical interventions for enhancing physical, psychological, social, and cognitive well-being by fostering social connectedness and reducing isolation among older adults. However, a gap in existing research is the limited understanding of the factors influencing the acceptability and feasibility of IGPs. Identifying these factors is essential for designing programs that better meet the needs of older adults. This study examines older adults’ perspectives on IGPs through semi-structured interviews with 24 participants (ages 65–104). Thematic analysis identified seven key factors that shape engagement: (1) Culture, (2) Knowledge and Experience Exchange, (3) Interest and Curiosity as Motivation, (4) Emotional Support, (5) Social Interaction, (6) Family and Community Support, and (7) Technology Integration. These factors influence how older and younger participants engage in IGPs, shaping their experiences through shared activities and reciprocal knowledge exchange. The findings emphasize the role of structured engagement in sustaining meaningful intergenerational interactions. While older adults show enthusiasm, younger participants often engage due to academic requirements, limiting intrinsic motivation. Differences in digital literacy create barriers to participation, particularly for older adults unfamiliar with technology. The study also found that varying expectations among older participants, limited institutional support, and the digital divide pose challenges to engagement. These insights enhance our understanding of intergenerational dynamics, guiding policymakers and program developers in designing interventions that promote healthy aging and strengthen intergenerational bonds.

